# Combined anterior cruciate ligament revision with reconstruction of the antero-lateral ligament does not improve outcome at 2-year follow-up compared to isolated acl revision; a randomized controlled trial

**DOI:** 10.1007/s00167-023-07558-x

**Published:** 2023-09-21

**Authors:** Ole Gade Sørensen, Peter Faunø, Lars Konradsen, Torsten Nielsen, Susanne Schaarup, Bjarne Mygind-Klavsen, Michael Krogsgaard, Martin Lind

**Affiliations:** 1 Department of Sports Traumatology, University Hospital of Aarhus, Aarhus, Denmark; 2 Section for Sports Traumatology, Bispebjerg, Frederiksberg University Hospital, Copenhagen, Denmark

**Keywords:** Anterolateral ligament, ACL revision, ALL outcomes, ACL surgery

## Abstract

**Purpose:**

It is essential to obtain rotational stability of the knee after anterior cruciate ligament reconstruction (ACL-R) and it is suggested that a supplementary reconstruction of the antero-lateral ligament (ALL-R) may supports this. Theoretically, ALL-R may be particularly advantageous to support revision of failed ACL-Rs. It was hypothesized that ACL revision combined with ALL-R will result in superior outcome compared to isolated ACL revision.

**Methods:**

The study was designed as a randomized controlled trial. Patients eligible for first time ACL revision were randomized to either isolated ACL revision (− ALL group) or ACL revision combined with a single-stranded allograft ALL-reconstruction (+ ALL group). Patient reported outcomes and function were evaluated at two-year follow-up by KNEES-ACL, KOOS, and Tegner activity scale. Objective knee laxity was evaluated at one-year follow-up using an instrumented Rolimeter test, the pivot shift test, and a manual Lachman test.

**Results:**

A total of 103 patients were enrolled with 49 patients randomized to the + ALL group and 54 patients in the − ALL group. There were no differences at baseline between groups regarding age, gender, body mass index, preoperative patient reported outcome scores and concomitant meniscus or cartilage injury. The ACL revision was performed with an allograft in 10 patients (20%) in the + ALL group and 8 patients (15%) in the -ALL group. At follow-up there was no significant difference between the groups in patient reported outcome scores and clinical knee laxity.

**Conclusion:**

Supplementary ALL-R does not improve subjective outcome of first time ACL revision at two-years and clinical knee stability at one-year follow-up compared to isolated ACL revision.

**Level of evidence:**

Level I.

## Introduction

ACL-R is a commonly performed orthopedic procedure that aims to restore the stability of the knee in patients who have sustained an ACL injury. However, residual rotational instability after ACL-R has been reported [1–3] which has led to an increased focus on the anterolateral aspect of the knee to control the internal rotation of the knee and thereby normalize rotational stability.

There are several standardized extra-articular stabilizing surgical procedures on the anterolateral side of the knee, including lateral extra-articular tenodesis (LET). These were once the primary procedures for the treatment of ACL deficiency [4, 5] and were later combined with intra-articular ACL reconstruction [6, 7].

The anterolateral ligament (ALL) was recently rediscovered and described in anatomical, biomechanical, and radiological studies [8–15]. Procedures to reconstruct the ALL have been reported by several authors [16–19] and can be regarded as alternative techniques to the traditional LET procedures in ACL surgery.

The outcome after ACL revision surgery is inferior to the outcome after primary ACL reconstruction in terms of subjective outcome, clinical knee stability, and the risk of further revision surgery [20, 21]. This has led to the suggestion that the addition of an anterolateral extra-articular procedure in ACL revision surgery could improve rotational knee stability and result in better subjective knee function [22].

The role of an anterolateral extra-articular procedure in ACL revision surgery has been previously investigated [23–33], but high-quality studies seem to be sparse.

The aim of this study was to evaluate the outcome after ACL revision with or without additional ALL reconstruction in a randomized controlled trial. The hypothesis was that combined ACL revision and ALL reconstruction would result in a superior outcome compared to isolated ACL revision.

## Materials and methods

The study was approved by the Danish ethical committee (1-10-72-324-15) and registered at clinicaltrial.gov (NCT02680821). The study was a randomized controlled trial performed at two departments that serve as referral centers for ACL revision patients. Enrollment and surgery were performed by senior surgeons between March 2016 and September 2019. ACL graft rupture was diagnosed by clinical examination and MRI. Inclusion criteria were patients between 18 and 50 years of age with an ACL-deficient knee and scheduled for first-time ACL revision were included. Exclusion criteria were language barriers and concomitant instability of the collateral ligaments or the posterior cruciate ligament.

### Patient characteristics

**Fig. 1:**
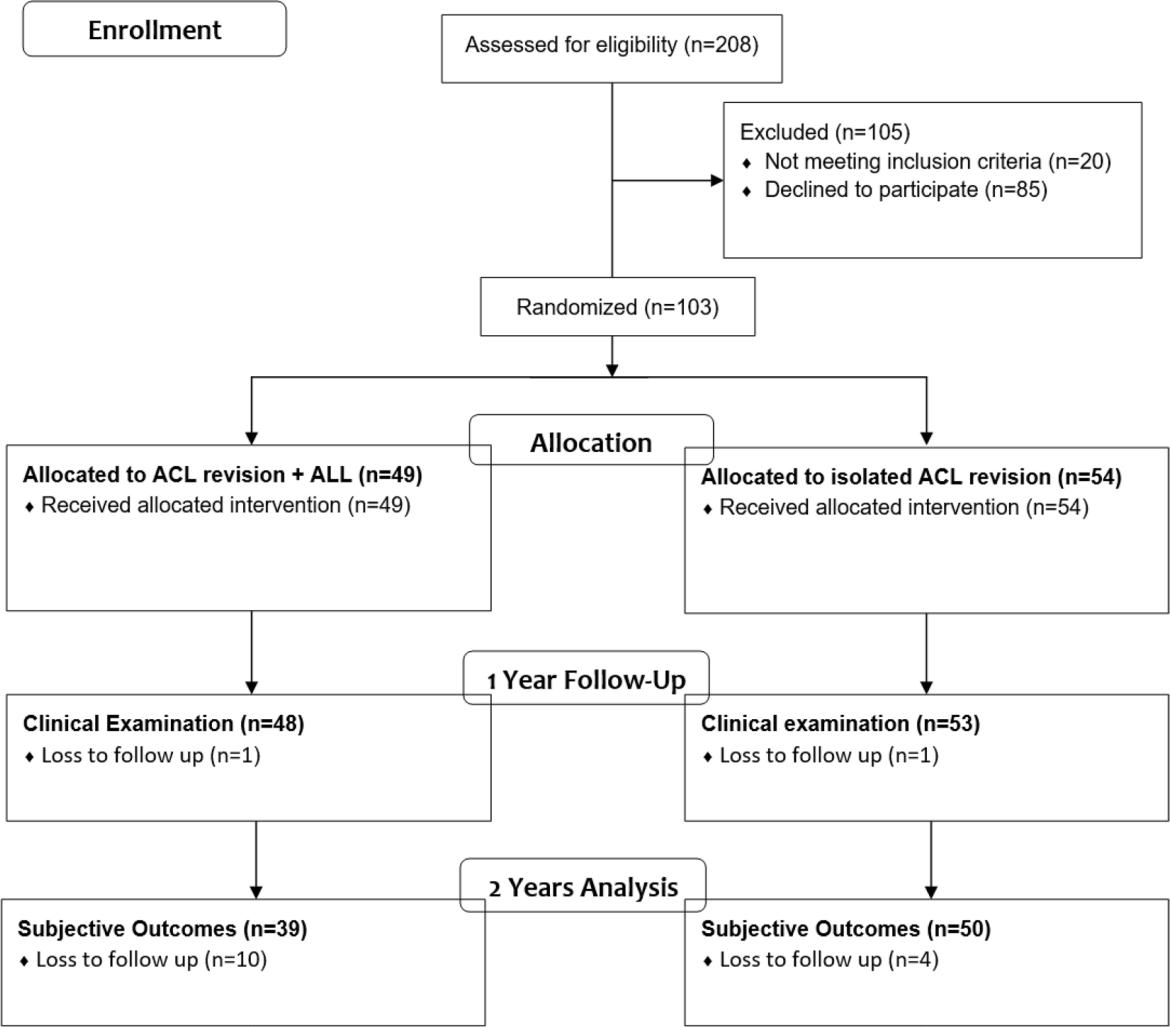
Consort flowchart

A total of 208 patients were assessed for eligibility. Eighty-five patients declined to participate, and 20 patients did not meet the inclusion criteria, leaving 103 patients for randomization (Fig. 1). Fourteen patients were lost to follow-up, leaving 89 patients for final analyses (Fig. 1).

There was a significantly better score in the KNEES-ACL Sport Physical subscale in the group that was randomized to ALL reconstruction, but otherwise, there was no significant difference between the groups at baseline (Table 1).

**Table 1:** Baseline characteristics

	+ ALL	- ALL	P-value
Number of patients	49	54	
Gender, n (%)			NS
Female	20 (41)	27 (50)	
Male	29 (59)	27 (50)	
Age, median (range)	28 (19–45)	26 (19–49)	NS
Months from graft failure to ACL revision, mean (range)	14 (2–41)	11 (2–46)	NS
Staged surgery, n (%)			NS
One stage	22 (45)	24 (44)	
Two stages	27 (55)	30 (56)	
ACL graft choice, n (%)			NS
Allograft	10 (20)	8 (15)	
Autograft	39 (80)	46 (85)	
Hamstring	3 (6)	5 (9)	
BPTB	23 (47)	28 (52)	
Quadriceps	13 (27)	13 (24)	
Meniscal status, n (%)			NS
No injury	21 (43)	28 (52)	
Injury without treatment	4 (8)	2 (4)	
Repair	8 (16)	10 (19)	
Resection	14 (29)	12 (22)	
Missing	2 (4)	2 (4)	
Cartilage status, n (%)			NS
Grade 0, no injury	19 (39)	17 (31)	
Grade 1–2	21 (43)	23 (43)	
Grade 3–4	8 (16)	8 (15)	
Missing	1 (2)	6 (11)	
Pivot shift, n (%)			NS
Grade 0	0 (0)	1 (2)	
Grade 1	18 (37)	13 (24)	
Grade 2	23 (47)	30 (56)	
Grade 3	5 (10)	6 (11)	
Missing	3 (6)	4 (7)	

*NS* non-significant, *n* number of patients, *BPTB* bone patella tendon bone

### Surgical procedure

After patient participation approval by written consent, the ACL revision procedure started with a knee arthroscopy to confirm the patient’s eligibility for the study. When one-stage ACL revision was possible, the patient was randomized per-operatively to ACL revision in combination with ALL reconstruction (+ ALL group) or isolated ACL revision (− ALL). Opaque envelopes with a note of which group the patient was randomized to were used for randomization. In cases of two-stage revision procedures, randomization was performed during the second stage procedure.

All surgical procedures were performed by senior surgeons. ACL revision was performed as a single-bundle anatomical reconstruction with the femoral tunnel drilled through an anteromedial portal. The ACL revision tunnel placement aimed for the anteromedial portion of the native ACL on both the femur and the tibia. The bone tunnels and tunnel apertures were assessed for acceptable diameter and placement. Acceptable tunnels were redrilled and reused for a one-stage revision procedure. Unacceptable tunnels were bone grafted and a two-stage ACL revision was performed. The choice of ACL graft and graft fixation took the primary ACL reconstruction into account, if necessary. Therefore, bone-patellar tendon-bone, quadriceps-tendon, and hamstrings-tendon autografts or allografts were used for the ACL revision (Table 1).

ALL reconstruction was performed in the + ALL group as a single-strand reconstruction using an iliotibial tract allograft. The graft was prepared as a 10 cm long and 1 cm wide strand. It was whip-stitched 2.5 cm in each end with a FiberWire^®^ braided composite suture size 0 (Arthrex, Naples FL). The lateral femoral epicondyle was palpated percutaneously. A 2-cm longitudinal skin incision was made, followed by blunt dissection of the iliotibial tract. The fibers of the iliotibial tract were divided. A K-wire was placed posterior and proximal to the lateral femoral epicondyle, as described by Dodds et al. [11]. A 6-mm cannulated drill was used to create a three cm deep bone tunnel. Another 2-cm longitudinal skin incision was made midway between the fibular head and Gerdy’s tubercle, followed by blunt dissection to the bone. A K-wire directed medially and distally was placed one centimeter below the joint line, and the second drill tunnel was created with a 6-mm cannulated drill to a depth of 3 cm. The iliotibial tract allograft was pulled into the femoral drill tunnel and fixated with a 6 × 23 mm PEEK^®^ interference screw (Arthrex, Naples FL). The ALL graft was tunneled deep to the iliotibial tract and superficial to the joint capsule toward the tibial bone tunnel. Tension was applied to the graft, which was fixated in the tibial drill tunnel with a 6 × 23 mm PEEK^®^ interference screw (Arthrex, Naples FL) in 20° of knee flexion and neutral rotation.

### Outcome measures

The Knee Numeric-Entity Evaluation Score (KNEES-ACL) [34, 35], the Knee Injury and Osteoarthritis Outcome Score (KOOS) [36], and the Tegner Activity Scale [37] were used for patient-reported outcome score evaluation. The KNEES-ACL consists of six subgroups and was developed for and by the involvement of patients with ACL injuries. The questionnaire has content and construct validity for this patient group [34, 35]. Each subgroup has a different maximum score. A low KNEES-ACL score reflects a better condition than a high score. The KNEES-ACL looseness subgroup was used as the primary outcome at the 2-year follow-up. Secondary outcomes were the other 5 subgroups of KNEES-ACL, the KOOS, and the Tegner Activity Scale [37] at the 2-year follow-up. Instrumented objective laxity evaluation of the knee was measured as a side-to-side difference with a Rolimeter® knee tester (Aircast Europe, Neubeuern Germany). The reliability of the Rolimeter was reported by Muellner et al.[38]. A manual pivot shift test was performed on both sides to evaluate rotational stability. The clinical measurements were carried out by independent investigators, but neither the patients nor the investigators were blinded to the treatment group.

### Postoperative rehabilitation

All patients used crutches for two weeks without bracing. The patients underwent a physiotherapist-guided rehabilitation program following surgery, in most cases for 3 months but with some regional differences. All patients were educated to continue knee stability self-training exercises after the guided physiotherapy period. Both departments serve as referral centers. The guided rehabilitation was carried out locally near the patient`s residence. Therefore, the duration of the guided rehabilitation period could be with regional differences.

### Statistical analysis

Normal distributed data were presented as mean (standard deviation [SD]) and other data as median (range). Student’s t-test and Wilcoxon rank sum test were used to test for differences in normally distributed and non-normally distributed data, respectively. Proportions were presented as numbers (n, %), and data were compared using Chi-square analysis. P-values < 0.05 were considered statically significant. Statistical analyses were computed using Stata version 17 (Stata Release 12, College Station, TX) and Excel version 2016.

### Sample size calculation

The initial sample-size calculation was based on an SD of 4.5 points for the primary outcome: subscale scores of the KNEES-ACL looseness domain. The minimal clinical important difference (MCID) was estimated to be 2.5 points. With a statistical significance (p) of 0.05 and a power of 80%, 51 patients were necessary in each group, and 102 patients were planned. Due to the loss of some patients to follow-up, a post-power sample-size calculation was made. The SD was smaller than assumed in the initial sample-size calculation, and the post-power calculation (with MCID = 2.5, p = 0.05, power = 80%, and SD = 3.3) showed that 28 patients were necessary in each group (56 patients in total).

## Results

### Primary endpoint

There was no difference between the two groups at the 2-year follow-up in relation to the primary endpoint, the KNEES-ACL Looseness subscale (Table 2).

**Table 2:** KNEES-ACL at baseline and 2-year follow-up

	+ ALL	− ALL	P-value		+ ALL	− ALL	P-value
	Baseline	Baseline			2 years FU	2 Years FU	
	(n = 49)	(n = 53)			(n = 39)	(n = 50)	
	Mean ± SD	Mean ± SD			Mean ± SD	Mean ± SD	
ADL	8.9 ± 5.8	10.2 ± 5.6	NS		6.9 ± 5.0	8.9 ± 5.3	NS
Range 0–24							
Psychosoc	6.6 ± 3.8	6.1 ± 4.2	NS		3.9 ± 3.4	5.1 ± 4.2	NS
Range 0–15							
Symptoms	6.6 ± 4.8	7.7 ± 5.1	NS		5.0 ± 3.9	6.4 ± 4.7	NS
Range 0–18							
Slackness	9.8 ± 4.9	9.7 ± 5.2	NS		7.4 ± 5.1	8.2 ± 5.2	NS
Range 0–21							
Looseness	6.6 ± 3.8	5.3 ± 3.5	NS		3.5 ± 3.3	4.1 ± 3.3	NS
Range 0–12							
Sport rec	12.6 ± 4.0	13.2 ± 4.4	NS		11.5 ± 5.2	11.7 ± 5.6	NS
Range 0–18							
Sport Phys	4.4 ± 3.7	6.0 ± 4.1	0.04		4.9 ± 4.0	5.1 ± 3.9	NS
Range 0–12							

*n* Number of patients, *FU* Follow-up, *SD* standard deviation, *ADL* Activity of daily living, *Psychosoc* Psychosocial, *Rec* Recreational, *Phys* Physical, *NS* not significant

### Secondary outcome

There was no difference between the two groups in scores from the five subdomains of KNEES-ACL that were secondary outcomes (Table 2) or in the KOOS subdomain outcome scores at the 2-year follow-up (Table 3). The median Tegner Activity Scale score at the 2-year follow-up was 4 (2–9) in the + ALL group and 4 (1–8) in the -ALL group.

**Table 3:** KOOS, Tegner, and objective outcome measures

Outcome measures	+ ALL	− ALL	P-value	+ ALL	− ALL	P-value
	Baseline	Baseline		2 years FU	2 years FU	
	(n = 49)	(n = 53)		(n = 39)	(n = 50)	
	Mean ± SD	Mean ± SD		Mean ± SD	Mean ± SD	
KOOS						
-Symptoms	70.8 ± 17.5	69.4 ± 16.9	NS	74.1 ± 1.51	73.5 ± 16.1	NS
-Pain	74.2 ± 15.7	72.7 ± 18.9	NS	82.7 ± 12.9	78.4 ± 17.5	NS
-ADL	81.9 ± 16.9	81.3 ± 15.9	NS	88.9 ± 10.1	86.4 ± 14.6	NS
-Sport	47.1 ± 26.3	45.3 ± 26.2	NS	62.2 ± 21.4	53.1 ± 25.9	NS
-QOL	36.3 ± 19.4	35.8 ± 16.4	NS	50.7 ± 18.2	44.9 ± 21.9	NS

	+ ALL	− ALL	P-value	+ ALL	− ALL	P-value
	Baseline	Baseline		1 year FU	1 year FU	
	(n = 46)	(n = 50)		(n = 47)	(n = 53)	
Tegner	4 (0–7)	4 (0–8)	NS	4 (2–9)	4 (1–8)	NS

	+ ALL	− ALL	P-value	+ ALL	− ALL	P-value
	Baseline	Baseline		1 year FU	1 year FU	
	(n = 46)	(n = 50)		(n = 47)	(n = 53)	
Side-to-side difference	5.6 ± 2.7	5.6 ± 2.7	NS	2.1 ± 1.6	2.2 ± 2.3	NS
Pivot shift	n (%)	n (%)		n (%)	n (%)	
Grade 0	1 (2)	2 (4)		21 (45)	33 (62)	1 (2)
Grade ≥ 1	45 (98)	48 (96)		26 (55)	20 (38)	45 (98)

*n* Number of patients, *Side-to-side difference* Difference in anterior laxity, *FU* Follow-up, *SD* standard deviation, *ADL* Activity of daily living, *QOL* Quality of life, *NS* not significant, Tegner score is given as median (range)

The measured side-to-side anterior laxity stability of the knee was significantly better for both groups at the 1-year follow-up compared to baseline, but there was no significant difference between the + ALL group and the -ALL group (Table 3). In total, 45% of the patients in the + ALL group had no side-to-side difference in the pivot shift grading at the 1-year follow-up compared to 62% in the -ALL group (Table 3), but the difference was not statistically significant (p = 0.11).

Within the first 2 years of follow-up, two patients in the + ALL group underwent ACL re-revision compared to one patient in the -ALL group. Re-surgery within the first 2 years in terms of partial meniscectomy, hardware removal, and cyclops removal was performed in eight patients in the + ALL group and seven patients in the -ALL group.

## Discussion

The most important finding of this study was that supplementary ALL-R in combination with ACL-revision did not result in improved subjective patient-reported outcomes or better clinical laxity and rotational knee stability compared to ACL-revision without ALL-R. Theoretically, the ALL-R should support internal rotational stability of the knee, but this could not be confirmed in the current study, in which there was actually a higher proportion of patients with a positive pivot shift in the + ALL group, although not statistically significant.

Although not statistically significant, the proportion of female male patients in the + ALL group was lower than in the − ALL group. Reported outcome after ACL revision based on gender is sparse in the literature but some evidence exists after primary ACL-R with studies reporting worse patient reported outcome in female patient compared to male patient after ACL reconstruction [39–41]. Theoretically, the male/female ratio in the present study could result in superior patient reported outcome measures in the + ALL group but no statistically significant differences were observed.

Clinical studies regarding the effects of combined ACL revision and ALL-R are sparse. Lee et al. [26] compared combined ACL-revision and ALL-R with isolated ACL revision in a retrospective design with a total of 87 patients and 3–4 years of follow-up. They used a tibialis tendon allograft for all ACL revision cases and a gracilis tendon allograft for the ALL-R in the combined group. In contrast to the present study, they reported a difference in the subjective IKDC score and the Tegner Activity Scale score in favor of the combined ACL revision + ALL-R procedure. No significant difference in the Lysholm score was observed. Furthermore, they found significantly better rotational stability of the knee in the combined group. The proportion of preoperative pivot shift grade 2–3 patients was higher in Lee’s study [26] than in the present study, which might explain the reported significant difference in the outcome scores. A retrospective study design was used in the Lee study with a cohort of isolated ACL revision patients compared to a combined ACL revision and ALL-R group. All the patient in the combined group had surgery after the isolated group, which could bias the superior outcome scores seen in the combined group. Louis et al.[28] reported the results of combined ACL revision and ALL-R in a multi-center study with a combined retrospective and prospective design and no control group. There were acceptable subjective outcome scores reported by the 349 patients. Most of the patients were graded with grade 2–3 pivot shifts prior to revision surgery. Only 1% of the patients had a clearly positive pivot shift postoperatively at follow-up, which is in vast contrast to the present study. Helito et al. [42] used a retrospective design to investigate the effect of an anterolateral procedure and ACL revision compared to isolated ACL revision in 174 patients. The extra-articular procedure in the combined group was either an ALL-R or a LET. They reported lower failure rate, superior objective outcome measures and a better Lysholm score in the combined group compared to the isolated ACL revision group.

Clinical outcomes after ACL revision combined with a LET procedure have been described in several studies. A review [43] included 12 studies concerning ACL revision combined with an anterolateral procedure. The study by Louis et al. [28] was included, leaving 11 studies with a total of 502 patients with combined ACL revision and a LET procedure. The clinical outcomes were acceptable, but the patient groups were highly heterogeneous, and the LET procedure was different in most of the included studies. The studies were a mix of retrospective and prospective cohort studies, and some were without control groups. The conclusion was that although ACL revision is often accompanied by a LET procedure, the results are conflicting, and there is a lack of high-level studies. Recently, Boksh et al. [44] investigated the effect of an anterolateral supplemental procedure in revision ACL-R in a meta-analysis. They included 10 comparative studies with a total of 793 patients [26, 31, 42, 45–51] and concluded that a lateral extra articular augmentation in ACL revision surgery could improve rotational stability, subjective IKDC scores, and reduce graft failure rate compared to isolated ACL revision.

Eggeling et al. [46] studied the effect of a modified Lemaire procedure in patients with low-grade anterior knee laxity and pivot shift grades 1–2 and found no effect of the LET in 27 patients who had a combined procedure compared to 55 patients with isolated ACL revision. In contrast, Alm et al.[45] evaluated the effect of a LET procedure in 75 ACL revision patients with high-grade anterior knee laxity and a grade 3 pivot shift test and found improved functional outcome and a lower degree of pivot shift in the LET + ACL revision group. Moreover, a significant reduction in ACL revision graft failure was seen in the combined group. As mentioned earlier, Lee et al. [26] showed significantly improved outcomes after ALL-R in a patient population with a high-grade pivot shift. Similarly, Louis et al. [28] demonstrated little residual rotational instability after ACL revision and ALL-R. Yoon et al. reported similar results. [51]. Perhaps, an anterolateral procedure should be considered in ACL revision patients showing a high-grade pivot shift preoperatively.

The effect of ALL-R in combination with ACL surgery has been investigated in several biomechanical studies with diverging results. A positive effect on both anterior laxity and internal rotation has been reported [52, 53] whereas others only show a significant reduction in internal rotation following ALL-R [54] with some risk of over-constraining at higher degrees of knee flexion [55]. Some studies have failed to show that combining ACL-R with ALL-R could restore native knee stability after combined lesions of ACL and the anterolateral capsule [56, 57]. However, biomechanical studies do not provide a uniform picture of the impact of ALL-R in combination with ACL surgery.

ALL-R reconstruction and LET in combination with ACL surgery have been compared in a number of biomechanical studies. Inderhaug et al. [58] investigated several different lateral procedures in combination with ACL reconstruction and found that supplementary ALL-R was not as effective as a modified Lemaire technique or a Macintosh procedure to restore native knee kinematics. In another study, the same group [59] found that a combination of ACL-R and a modified Lemaire procedure could restore pre-injury knee kinematics at all graft-fixation angles. Supplemental ALL-R resulted in equally good outcomes with fixation at zero degree of flexion but with inferior outcomes at higher flexion angles. Spencer et al. [60] found similar results for a combined anterolateral procedure and ACL-R. The modified Lemaire technique was reported to be superior in restoring anterior laxity and rotational stability compared to ALL-R. In contrast, some studies have reported similar improvements in internal rotation and anterior knee laxity after ACL-R combined with either anterolateral tenodesis or ALL-R [61, 62]. Similar findings were reported with acceptable restoration of the internal rotation at low knee flexion angles but with a tendency of over constraining at higher flexion angles for both the ALL-R and the LET [62, 63].

ACL revision patients show a high grade of diversity in knee pathology, and ALL-R or a LET procedure might be just one of several possible supplemental procedures that can address abnormal soft tissue and bony anatomy [64, 65]. Other procedures include meniscal transplantation, cartilage repair, and slope-changing osteotomy. The literature on lateral surgical procedures is still very heterogeneous regarding surgical techniques and patient cohorts, and most are low-level quality studies, which makes the decision of an anterolateral procedure in ACL revision patients difficult. Therefore, high-quality studies are needed to identify the right indication for anterolateral procedures in combination with ACL revision reconstruction. To our knowledge, the present study is the first randomized trial addressing the effect of ALL-R as a supplement to ACL revision reconstruction, and the results of this level 1 study could not confirm that ALL-R provides clinical benefit.

Getgood et al. [66] showed reduced graft failure in combined ACL reconstruction and LET compared to isolated ACL reconstruction in 618 young patients with high-grade pivot shift and/or hyperlaxity returning to pivoting sports. Similarly, future studies could attempt to identify subgroups of ACL revision patients who could benefit from an anterolateral procedure.

Due to the loss of patients to follow-up, this study did not meet the necessary number of patients defined in the a priori sample size calculation. This carries a risk of type 2 errors. However, the actual standard deviation (SD) of the KNEES-ACL score was lower than expected, and a post hoc sample size calculation showed that only approximately 30 patients were needed in each group.

The ALL-R resulted in two extra incisions on the lateral side, which means that the patients were not blinded regarding the procedure. The objective measurements were performed by an independent investigator with no influence on patient enrollment, randomization, or surgical procedures, but the investigator was not blinded in relation to which group each patient belonged to. The lack of blinding normally leads to a positive effect for the intervention group. However, in this study, the intervention group was not superior at any endpoint. The pivot shift assessment is based on the skills of the examiner, which could bias the results of rotational instability in this study. Regional differences of the physiotherapist guided rehabilitation period were possible as described in the method section. Hopefully, the differences were divided equally between groups because of the randomized study design but the decentralized rehabilitation of patients could carry a potential bias.

## Conclusion

Combined ALL-R and ACL revision does not improve subjective outcome at the 2-year follow-up and objective outcome at the 1-year follow-up compared to isolated ACL revision.

## Data Availability

Data can be provided.
